# Blood–Brain Barrier (BBB)-Penetrable Androgen Receptor (AR) Degrader as a Potential Therapeutic Agent for Glioblastoma

**DOI:** 10.1021/acsptsci.5c00821

**Published:** 2026-04-06

**Authors:** Xiaotong Zhao, Yaxin Li, William R. Martin, Fatma Salem, Ruchitha Korem, Soha Bolbol, Garrett Worden, Maheen Sajid, Alia Al-Rousan, Blake Wilt, Wenjing Zhang, Lyubomyr Turchyn, Christopher Hubert, Liqun Ning, Justin D. Lathia, Aimin Zhou, Bin Su

**Affiliations:** † Department of Chemistry, Center for Gene Regulation in Health and Disease, College of Sciences and Health Professions, 2564 Cleveland State University, 2121 Euclid Ave., Cleveland, Ohio 44115, United States; ‡ Genomic Medicine Institute, Cleveland Clinic Genome Center, Lerner Research Institute, Cleveland Clinic, Cleveland, Ohio 44195, United States; § Animal Research Facility, 2564 Cleveland State University, 2121 Euclid Ave., Cleveland, Ohio 44115, United States; ∥ Department of Biochemistry, School of Medicine, 2546 Case Western Reserve University, 10900 Euclid Ave., Cleveland, Ohio 44106, United States; ⊥ Department of Mechanic Engineering, College of Engineering, 2564 Cleveland State University, 2121 Euclid Ave., Cleveland, Ohio 44115, United States; # Department of Cancer Sciences, Cleveland Clinic Research, Cleveland, Ohio 44195, United States; ∇ Department of Molecular Medicine, Cleveland Clinic Lerner College of Medicine of Case Western Reserve University, Cleveland, Ohio 44195, United States; ○ Case Comprehensive Cancer Center, Cleveland, Ohio 44195, United States; ◆ Department of Biological, Geological, andEnvironmental Sciences, Center for Gene Regulation in Health and Disease,College of Arts and Sciences, Cleveland State University, 2121 Euclid Ave., Cleveland, Ohio 44115, United States

**Keywords:** glioblastoma, androgen receptor, sex disparity, BBB, protein degradation

## Abstract

Androgen receptor (AR) contributes to the progression of glioblastoma (GBM), which is consistent with the sex difference in GBM, which has a higher incidence in males than in females. Therefore, targeting AR is a potential therapeutic approach for GBM treatment. However, AR mutation commonly occurs in GBM, which makes conventional AR antagonists less effective. AR degraders abolish AR at the protein level regardless of the mutation status of AR, which makes it a better strategy in GBM. Compound **A** is an analog of the cyclooxygenase-2 (COX-2) inhibitor Nimesulide. Mechanistically, compound **A** targets HSP27, disrupts the HSP27-AR complex, and thereby promotes AR degradation in GBM cells at 1 μM, leading to inhibition of AR-overexpressing GBM cell growth with IC_50_s around 0.2 μM. In a GBM patient-derived cell line, DI318, compound **A** (1 μΜ) also significantly decreases AR protein levels. The compound significantly inhibits GBM xenograft growth at 20 mg/kg and does not cause toxicity in mice up to 200 mg/kg. Pharmacokinetic studies reveal that compound **A** has a half-life (*t*_1/2_) of 3.11 h and a BBB penetration of 52%, which is even higher than the standard chemotherapy Temozolomide. These results suggest that the AR degrader has great potential as a novel GBM treatment.

## Graphical abstract

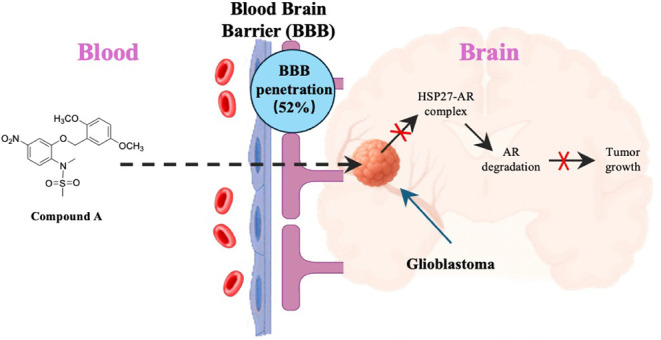

Glioblastoma (GBM) stands as the most prevalent primary brain tumor and aggressive form of brain cancer. Despite advances in surgical techniques and the standard treatment regimen combining Temozolomide (TMZ) chemotherapy with radiotherapy, the prognosis for GBM patients remains poor.^1–3^ TMZ, an alkylating agent, is still the key GBM chemotherapy due to its strong ability to cross the blood-brain barrier (BBB).^4^ However, both innate and acquired resistance to TMZ are frequent among GBM patients, severely limiting therapeutic success.^3,5,6^ There is an urgent need to identify new molecular targets and develop more effective therapeutic strategies. Interestingly, GBM incidence is notably higher in men compared to women, and male patients also tend to experience worse outcomes.^1,7,8^ Research has uncovered the molecular differences underlying these sex differences in GBM. These observations suggest a possible role of sex hormone pathways in GBM pathogenesis.^9^ Further investigations have revealed that androgen receptor (AR) is overexpressed in GBM, with androgens promoting tumor progression. The elevated expression of AR in GBM correlates with the observed sex differences in disease rates.^10–13^ Consequently, targeting AR has emerged as a novel therapeutic approach, and clinical trials are currently evaluating Seviteronel, an AR inhibitor, in patients with AR-overexpressing GBM.^14^ However, a significant challenge has surfaced: approximately 30% of GBM cases with AR overexpression have AR mutations, potentially undermining the effectiveness of AR antagonist therapies.^15^ Thus, it becomes critical to explore alternative strategies to suppress AR activity.

One promising avenue involves targeting the heat shock protein 27 kDa (HSP27), a molecular chaperone that stabilizes AR among other client proteins. HSP27 enhances the stability of AR, helping cancer cells evade treatment-induced death.^16,17^ Given this relationship, HSP27 presents an attractive target for disrupting the AR signaling axis in GBM. Unlike other chaperones, HSP27 operates via an ATP-independent mechanism, rendering it resistant to inhibitors such as geldanamycin derivatives that target ATP-binding sites.^18–23^ Therefore, strategies aiming at the mRNA level, such as short interfering RNA (siRNA) or antisense oligonucleotides (ASO), have been employed to silence HSP27 expression, subsequently reducing AR levels.^16–19^ Nonetheless, the presence of the BBB presents a significant hurdle for these biological approaches, as both ASOs and siRNAs show poor penetration into the brain, limiting their application in GBM therapy. Thus, small-molecule AR degraders that can efficiently cross the BBB are highly desirable for advancing GBM treatment.

In our previous studies, we developed AR degraders derived from the Cyclooxygenase-2 (COX-2) inhibitor Nimesulide as a starting scaffold (Figure 1).^24,25^ These compounds demonstrated potent *in vitro* and *in vivo* activity against AR-overexpressing GBM. Unfortunately, their BBB penetration was minimal, with crossing rates below 5%, restricting their therapeutic promise.^24–26^ In the current study, we conducted a systematic evaluation of the structural intermediates of these compounds and unexpectedly discovered a new lead molecule that displayed strong anti-GBM activity both *in vitro* and *in vivo*. Remarkably, this new compound showed a BBB penetration rate of 52%, surpassing even TMZ, which achieves a 20% rate.^4^ Furthermore, the new AR degrader exhibited no observable toxicity in mice, even at doses ten times higher than the effective therapeutic dose. Overall, our findings suggest that this structurally simplified AR degrader holds significant promise as a novel therapeutic candidate for AR overexpressing GBM.

**Figure 1:**
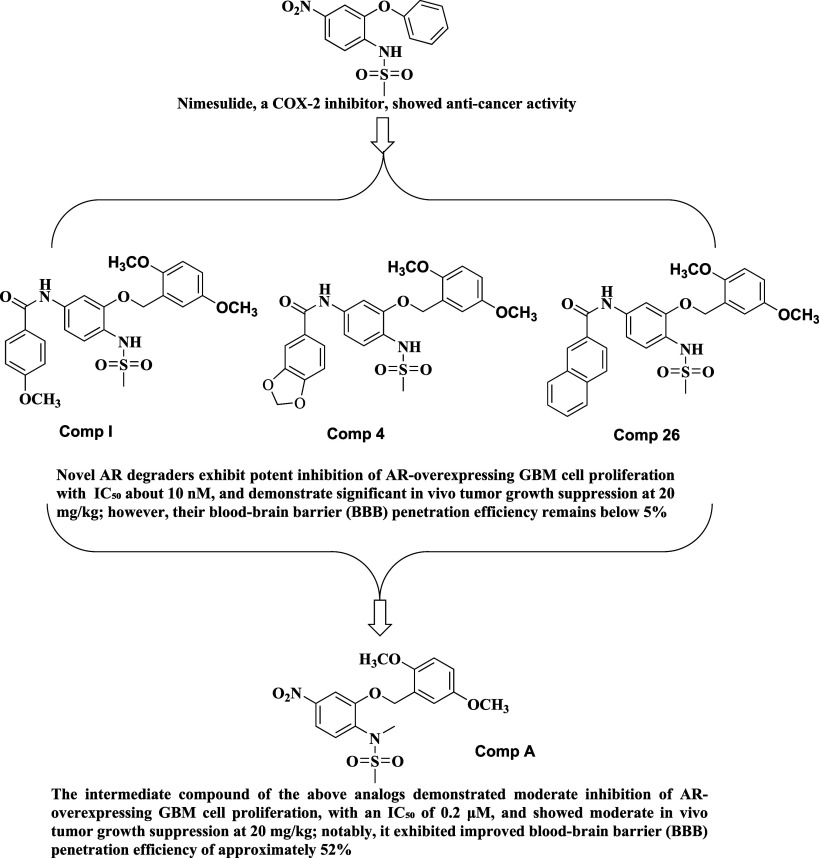
A structure reverse selection to identify AR degrader with better BBB penetration.

## Results and Discussion

### From Nimesulide to AR Degrader against GBM

Studies have shown that AR is highly expressed in a subset of GBM patients and is associated with a higher incidence of the disease in men compared to women.^9–13^ Given the frequent mutations of AR in GBM, traditional AR antagonists may be less effective in treating this cancer.^7,15^ Targeting and eliminating AR protein entirely offers a greater advantage, as this approach would remove both the wild-type and mutated forms of AR. To pursue this strategy, we developed a novel approach aimed at inducing AR degradation by inhibiting HSP27 in GBM cells, since AR is a well-established client protein of HSP27 and relies on HSP27 for its stability.^24,25^

To develop the most promising compounds targeting GBM, we have investigated the *in vitro* activity of the AR degradation of HSP27 inhibitors designed from our previous studies (Figure 1).^24,25^ We have identified three potent AR degraders that exhibited low nanomolar IC_50_ values against the proliferation of AR-overexpressing GBM cells. These compounds also effectively induced AR degradation in GBM cells, with comparable potency observed at concentrations of 50–100 nM. In the *in vivo* study, all three compounds significantly inhibited tumor growth in xenograft models at a dose of 20 mg/kg, and demonstrated no toxicity even at substantially higher doses. However, their ability to penetrate the blood–brain barrier (BBB) was limited, with brain/plasma ratios below 5% observed in pharmacokinetic studies.^24,26^

We hypothesize that a smaller scaffold with higher ligand efficiency may increase the BBB crossing rate.^27^ Therefore, we investigated the synthetic intermediate compound **A** for the synthesis of the three AR degraders developed previously. The compound is significantly smaller than the three identified AR degraders (Figure 1), and shows only one tenth potency to inhibit the proliferation of T98G and U87 GBM cells compared to the three previous AR degraders (Table 1).^24,25^

**Table 1:** Growth Inhibitory Effects of the Four Compounds in GBM Cells

**Compounds**	**T98G (nM)**	**U87 (nM)**
Comp I	2.0 ± 0.6	4.8 ± 1.6
Comp 4	35 ± 11	120 ± 45
Comp 26	23 ± 7	67 ± 25
Comp A	240 ± 60	850 ± 200

We further determined whether the intermediate still decreases AR protein levels in GBM cells. The results in Figure 2 shows that compound **A** could significantly downgrade AR protein level in T98G and U87 cells. Moreover, it could downregulate mutated AR (AR-V7) in T98G cells as well. So far, compound **A** could inhibit AR overexpressing GBM cell proliferation potently and downgrade AR protein level effectively.

**Figure 2:**
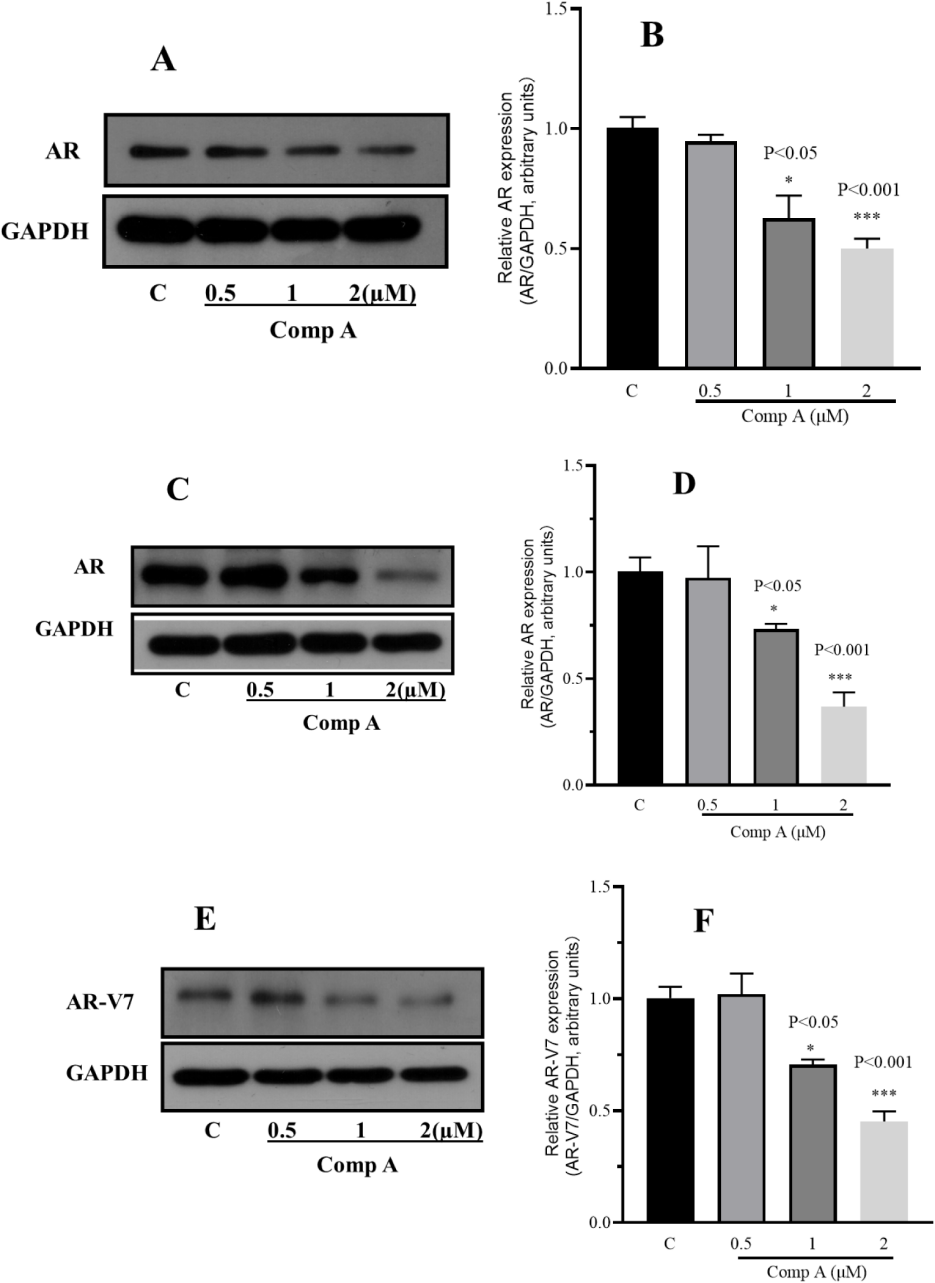
Compound **A** abolishes AR/mutated AR in GBM. U87 (panels A and B) and T98G (panels C–F). AR and AR-V7 protein expression after compound treatment was evaluated by Western blotting. The results shown represent one of three independent experiments with corresponding densitometric analysis. Values are presented as Mean ± SD (*n* = 3). Statistical differences relative to the DMSO control were assessed using an unpaired *t* test (*p < 0.05, ***p < 0.001).

### Compound **A** Inhibits Chaperone Activity of HSP27 and Decreases pHSP27

Previously identified AR degraders worked through HSP27 inhibition to decrease AR function; therefore, we determined if compound **A** maintains the same molecular mechanism. To examine the *in vitro* chaperone activity, insulin was used as a model substrate protein to mimic the protein aggregation and HSP27 served as the chaperone to prevent the aggregation.^24,25^ In this model, dithiothreitol (DTT) was used to denature insulin, which induced insulin β chain to aggregate. We used α-Crystallin which is the chaperone function domain of HSP27 to perform the chaperone assay. In the presence of the chaperone protein, the aggregation of insulin can be suppressed due to the formation of stable complexes between the chaperone and the unfolded β chain. Insulin aggregation was monitored by measuring absorbance at 400 nm. The ability of compound A to modulate the *in vitro* chaperone activity of HSP27 was assessed with or without compound **A** by using the method. As shown in Figure 3, α-Crystallin effectively suppressed DTT-induced insulin aggregation, demonstrating its chaperone activity. Compound **A** alone did not affect insulin aggregation under the same conditions. However, when compound **A** was coincubated with α-Crystallin, a notable increase in insulin aggregation was observed under DTT, indicating a reduction in α-Crystallin’s chaperone function. This was reflected by a higher absorbance and an upward shift of the aggregation curve compared to α-Crystallin alone. These results demonstrate that compound **A** inhibits the *in vitro* chaperone activity of HSP27.

**Figure 3:**
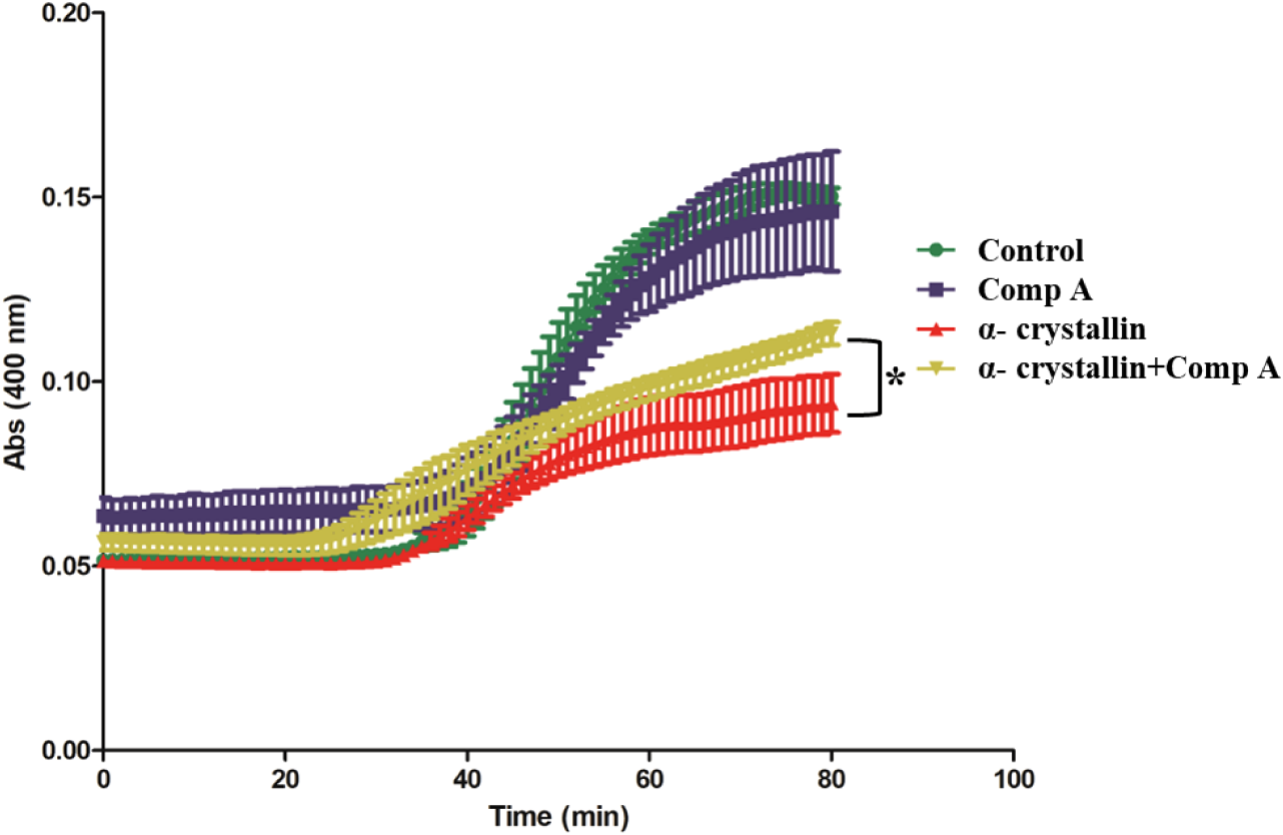
Inhibitory effect of compound **A** on the chaperone activity of HSP27. α-Crystallin, the functional chaperone domain of HSP27, was used to suppress DTT-induced insulin aggregation in the presence or absence of compound **A**. The kinetics of insulin aggregation induced by DTT were monitored either without a chaperone protein or in the presence of the chaperone with or without compound **A**. Reaction mixtures containing insulin and DTT, together with additional assay components when indicated, were incubated at 37 °C for 80 min, and aggregation was monitored by measuring absorbance at 400 nm. At the tested concentrations, compound **A** did not interfere with the interaction between insulin and DTT. The results shown are representative of three independent experiments. Each curve was obtained from triplicate measurements, and the averaged values were used to generate the curves. Statistical analysis was performed on the final absorbance values using an unpaired *t*-test (*p < 0.05, compound **A** vs control without compound **A**).

To further investigate the molecular interaction between compound **A** and HSP27, we examined the phosphorylation status of HSP27 following compound treatment. Basal phosphorylation levels of HSP27 in both U87 and T98G cells were low and undetectable with the current antibody, necessitating heat shock to enhance phosphorylation for reliable detection. As shown in Figure 4, treatment with compound **A** at 0.5 and 1 μM significantly inhibited phosphorylation of HSP27 at the Ser78 site in both cell lines. In contrast, no change was observed in Ser82 phosphorylation (data not shown). These findings suggest that compound **A** not only inhibits the chaperone activity of HSP27 but also suppresses its phosphorylation at Ser78.

**Figure 4:**
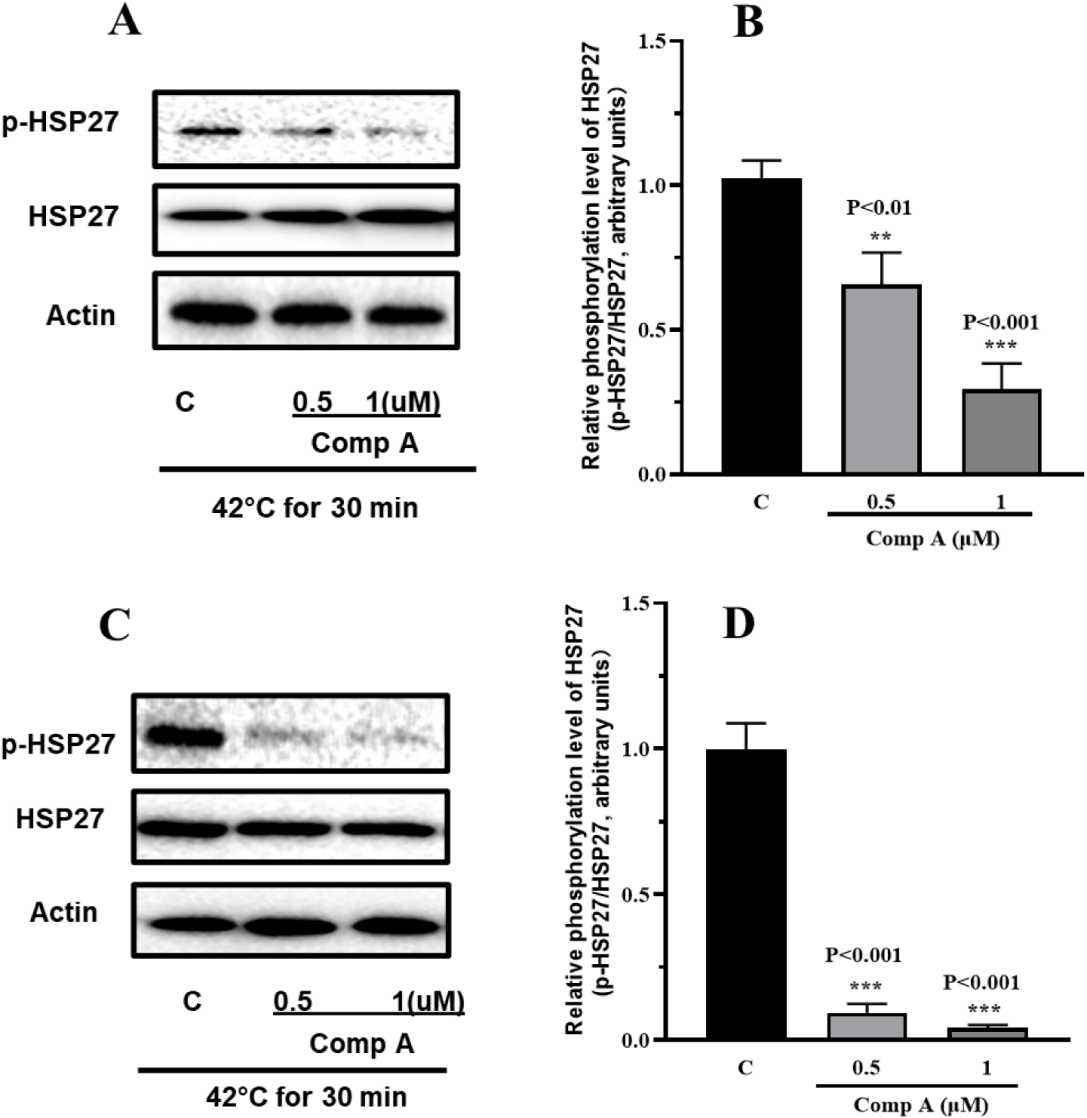
Inhibition of HSP27 phosphorylation by compound **A**. T98G (panels A and B) and U87 (panels C and D) glioblastoma cells were treated with compound **A** followed by heat shock at 42 °C. The phosphorylation levels of HSP27 were analyzed by Western blot. The experiment was independently repeated three times, and representative blots along with quantitative analysis are presented. Data are shown as mean ± SD (*n* = 3). Statistical significance was determined using an unpaired *t*-test (**p < 0.01, ***p < 0.001 compared to the control group).

Based on the inhibition of compound **A** to HSP27 phosphorylation, molecular docking was conducted using the crystal structure of HSP27 (PDB ID: 6DV5).^25,28^ HSP27 forms a multimeric assembly in which the biologically relevant phosphorylation region lies at the interface between two monomers, with residues S78 and S82 playing key roles. Docking analysis suggested that compound **A** can occupy the region near S78 through a hydrogen bond formed between the serine residue and the oxygen atom of the sulfonamide group (Figure 5). Such interaction may interfere with phosphorylation of HSP27 and subsequently disrupt its interaction with client proteins. These docking results agree with the observed inhibition of HSP27 phosphorylation by compound **A**. Beyond its role in AR stabilization, HSP27 is involved in cellular stress adaptation and has been implicated in therapeutic resistance in multiple tumor types.^17–19^ In GBM specifically, AR signaling has been reported to participate in pro-survival and redox-regulatory pathways that contribute to TMZ resistance.^5,6^ Given that compound **A** disrupts the HSP27-AR complex and promotes AR degradation, it is mechanistically plausible that this compound could potentially attenuate stress-protective signaling associated with TMZ resistance. Therefore, compound **A** may sensitize TMZ-resistant GBM cells to TMZ, a possibility that warrants direct experimental investigation.

**Figure 5:**
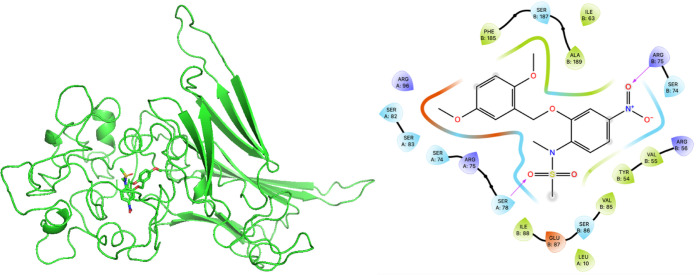
Molecular docking of compound A to HSP27. (Left panel) Molecular docking model illustrating the predicted binding pose of compound **A** near the phosphorylation site of HSP27­(PDB ID: 6DV5). (Right panel) Two-dimensional ligand interaction diagram showing key contacts between compound **A** and residues within the HSP27 binding pocket, where Ser78 is sterically hindered by the sulfonamide group, potentially preventing its phosphorylation.

Taken together, these findings support a model in which compound **A** targets HSP27, inhibits its phosphorylation at Ser78, disrupts the HSP27-AR complex, and promotes AR degradation, ultimately suppressing GBM tumor growth. A schematic illustration summarizing the proposed mechanism of action is shown in Figure 6.

**Figure 6:**
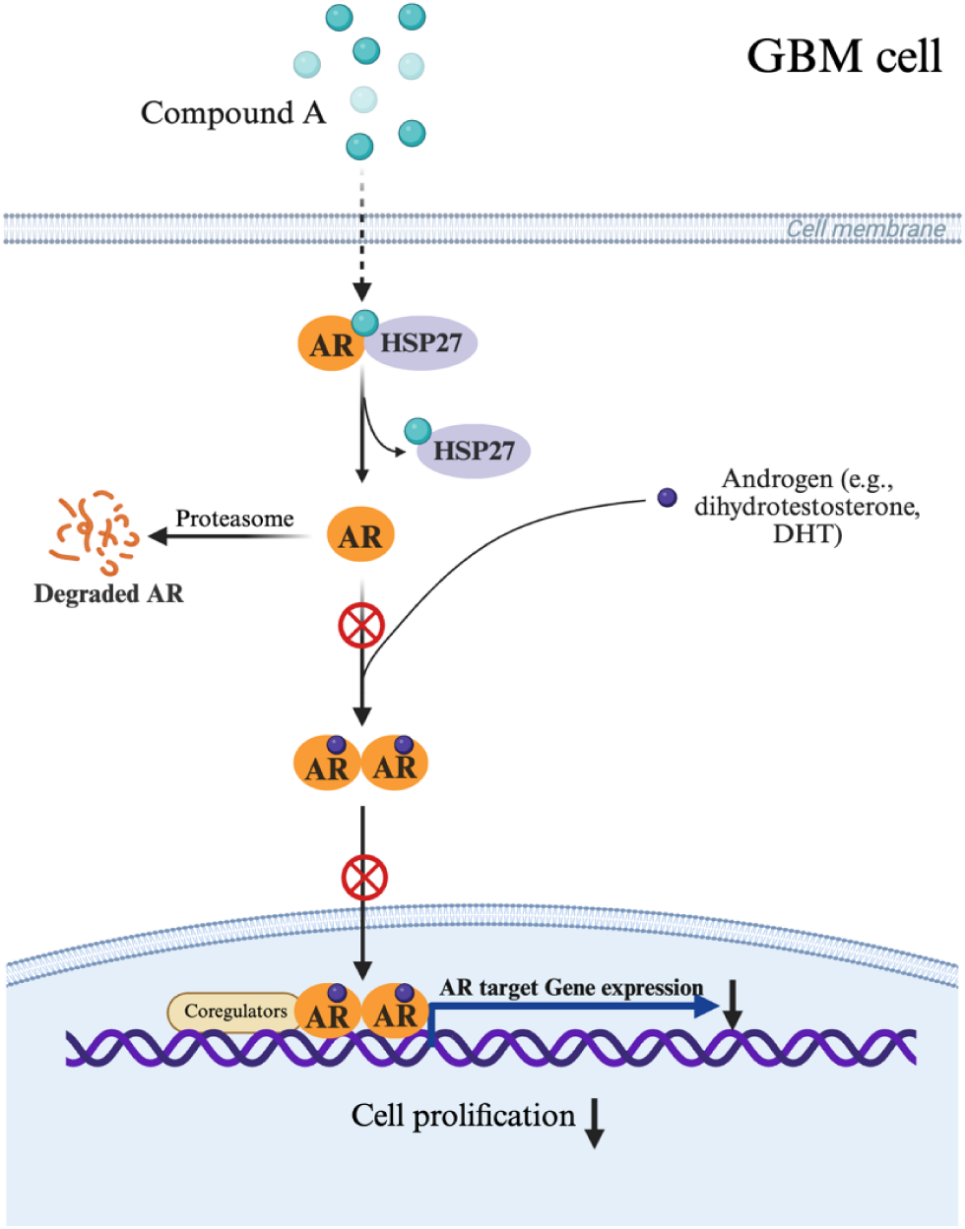
Schematic illustration of compound **A** targeting effect to HSP27, disruption of the HSP27-AR complex, and subsequent AR degradation in GBM (Created with BioRender.com).

### Compound **A** Significantly Reduces the Growth of the Xenograft Tumor and AR Levels

To assess the in *vivo* antitumor activity of compound **A**, a xenograft model was generated by subcutaneous implantation of U87 glioblastoma cells into both flanks of nude mice. Once tumors reached approximately 200 mm^3^, compound **A** was administered via intraperitoneal (IP) injection every 2 days for a period of 2 weeks. Tumor volume and body weight were monitored throughout the treatment period. As shown in Figure 7C, compound **A** treatment had no significant effect on body weight, indicating minimal systemic toxicity. In contrast, tumor volume was significantly reduced in the compound **A** treated group compared to the DMSO control group (*p* < 0.05; Figure 7B). Although a reduction in tumor weight was also observed in the compound **A** group, the difference was not statistically significant (Figure 7D). To determine whether the antitumor effect was associated with AR modulation, AR expression levels in tumor tissues were assessed via Western blotting (Figure 7E). A significant reduction in AR expression was observed in tumors from the compound A treated mice compared to controls (Figure 7F). These findings demonstrate that compound **A** exhibits strong *in vivo* antitumor activity and supports its potential as a promising therapeutic candidate for AR-overexpressing GBM.

**Figure 7:**
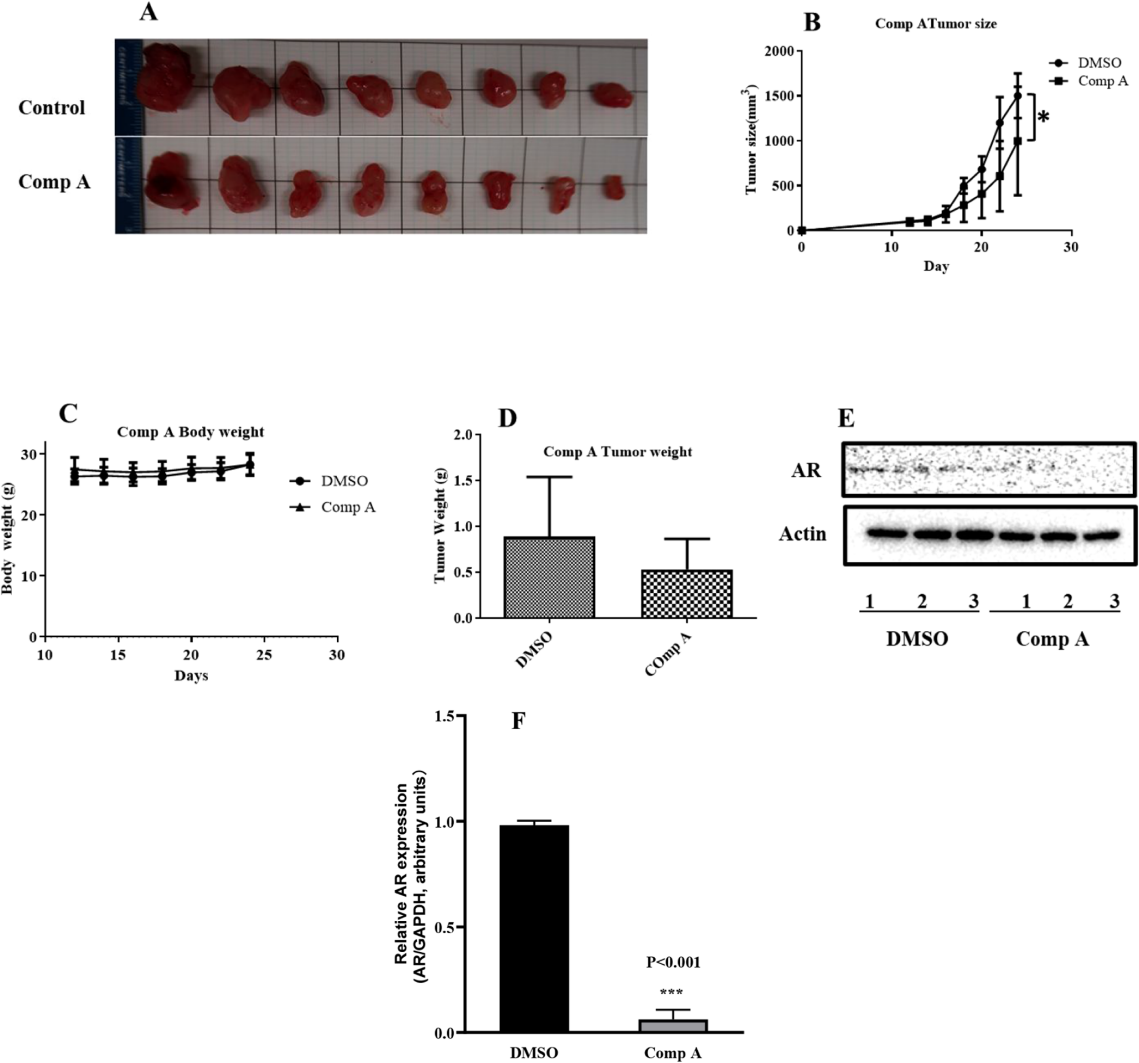
*In vivo* antitumor efficacy of compound **A** in a human GBM xenograft model. U87 cells (5 × 10^6^) were subcutaneously implanted into nude mice. The mice were randomly divided into a DMSO control group and a compound **A** treatment group. Tumor-bearing mice received compound **A** (20 mg/kg) by IP injection. Recovered tumors are shown in panel A (*n* = 8). Tumor volume is presented in panel B (*n* = 8). Body weight of the mice is shown in panel C (*n* = 4). Tumor weights are summarized in panel D (*n* = 8), and the data are expressed as Mean ± SD. AR expression in tumor tissues was analyzed by Western blot, with representative images and quantification shown in panels E and F (*n* = 3).

### Compound **A** Distribution in Brain Tissue

Compound **A** markedly inhibited GBM tumor growth in the mouse xenograft model. Nevertheless, it remained uncertain whether the compound could penetrate the BBB. Therefore, pharmacokinetic analysis of compound **A** in mouse plasma and brain tissues was performed by LC-MS/MS. Mice were administered the compound and sacrificed at predetermined time points for sample collection. Serum was prepared from blood samples, and brain tissues were collected following perfusion. The concentrations of compound **A** detected in serum and brain tissue over time are shown in Figure 8, and the pharmacokinetic parameters are summarized in Table 2 (Mean ± SD). The results demonstrate rapid systemic absorption and distribution of compound **A**. The apparent half-life (**t**_**1/2**_) of compound **A** in plasma was 3.11 h. However, sustained exposure was observed in brain tissue. This pattern likely reflects the complex distribution dynamics of compound **A** within the central nervous system. Brain drug concentrations were determined by the interplay between plasma–brain concentration gradients, bidirectional transport across the blood–brain barrier (BBB), tissue binding within the brain parenchyma, and physicochemical properties such as lipophilicity. Together, these factors can result in prolonged apparent brain retention relative to plasma.

**Figure 8:**
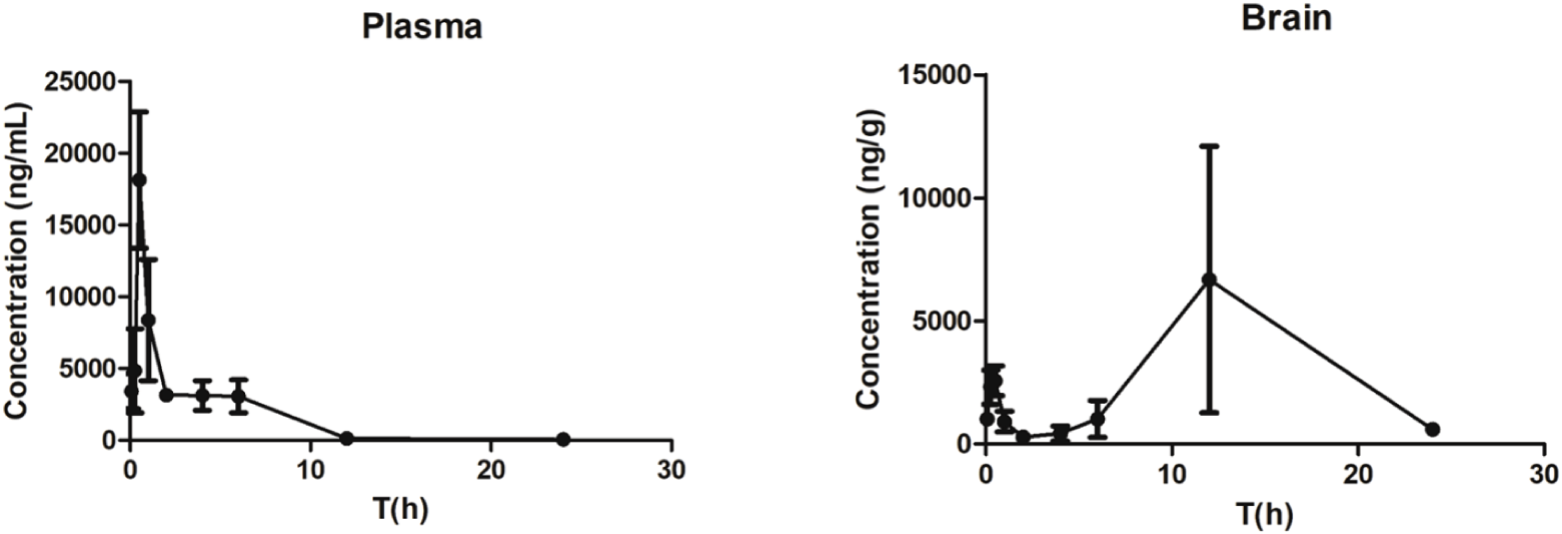
Concentration–time profiles of compound **A** in mouse plasma and brain tissue following IP administration (Mean ± SD, *n* = 4).

**Table 2:** Pharmacokinetic Parameters of Compound **A** in Mice Determined by Non-Compartmental Analysis After IP Administration (*N* = 4, Mean ± SD)^a^

	**Compound A**	
**Pharmacokinetic parameters**	**Plasma**	**Brain**
***t*** 1/2	3.11 ± 0.70 (h)	N/A
**C**_**max**_	18.15 ± 94.82 (μg/mL)	6.68 ± 10.82 (μg/g)
**AUC**_**0–24h**_	9.27 ± 4.02 (μg^.^h/mL)	4.90 ± 6.33 (μg^.^h/g)
**AUC**_**0‑∞h**_	9.68 ± 3.69 (μg^.^h/mL)	5.01 ± 6.58 (μg^.^h/g)
***R*** ^2(*)^	0.8187	N/A

a *“*N/A” not able to calculate, (*) terminal phase linear regression.

Importantly, the measured brain *C*_max_ of compound **A** was 6.68 ± 10.82 μg/g, corresponding to an estimated concentration of approximately 17 μM in brain tissue (based on the compound **A** molecular weight of 396 g/mol). Although total brain concentration does not necessarily represent the unbound fraction, this estimated exposure is within or above the concentration range at which compound **A** inhibited HSP27 Ser78 phosphorylation in cellular assays (0.5–1 μM). These findings suggest that pharmacologically relevant target engagement of the HSP27–AR axis may be achievable *in vivo*.

Pharmacokinetic analysis also showed an AUC of 9.68 μg·h/mL for compound **A** in plasma, whereas the AUC in brain tissue reached 5.01 μg·h/g, corresponding to roughly 52% of the plasma exposure. The detection of substantial drug levels in the brain suggests that compound **A** can penetrate the BBB and accumulate within brain tissue. The BBB crossing efficiency of compound **A** is even better than TMZ (20% permeability).^4^ This pronounced brain exposure prompted further examination of structural features that may underlie the enhanced permeability. Compared with our previously optimized AR degraders, compound **A** is structurally simplified and possesses a substantially reduced molecular weight: compound **A** (396 Da) vs Comp I, Comp 4, and Comp 26 (486 Da, 500.52 Da, and 506.57 Da, respectively, Figure 1). Reduced molecular weight is widely recognized as a favorable parameter for blood–brain barrier penetration and may facilitate passive diffusion across the BBB.^29^ In contrast, the larger and more structurally complex degraders previously reported exhibited minimal BBB penetration (<5%). Collectively, these observations suggest that scaffold simplification—at least in part through lowering molecular weight—likely contributed to the enhanced BBB permeability observed for compound **A**.

### Compound **A** Shows Great Therapeutic Index

To further evaluate the toxicity profile of compound **A**, C57BL/6 mice were treated with a high dose of 200 mg/kg daily for 10 consecutive days. During the treatment period, no signs of toxicity, including dehydration, acute pain, or distress, were noted in the mice. Body weight was monitored daily, and no significant differences in growth rate were noted between the compound **A** treated group and the control group. Hematological analysis revealed no significant alterations in blood parameters when compared to controls, with the full results presented in Table 3.

**Table 3:** Hematological Parameters of C57BL/6 Mice Treated with Compound **A** (200 mg/kg/day) for 10 Days

	Values (mean)		
Parameters (unit)	Ctrl	Comp A	Reference values
WBC (10^3^/μL)	5.66 ± 3.79	5.84 ± 1.98	0.80–10.6
Neu (10^3^/μL)	0.82 ± 0.74	1.44 ± 0.65	0.23–3.60
Lym (10^3^/μL)	4.28 ± 2.62	4.02 ± 1.33	0.60–8.90
Mon (10^3^/μL)	0.38 ± 0.36	0.18 ± 0.03	0.04–0.40
Eos (10^3^/μL)	0.16 ± 0.08	0.17 ± 0.01	0.00–0.51
Bas (10^3^/μL)	0.02 ± 0.02	0.03 ± 0.02	0.00–0.12
Neu (%)	12.50 ± 3.99	23.77 ± 4.10	6.50–50.0
Lym (%)	78.38 ± 5.56	68.93 ± 1.85	40.0–92.0
Mon (%)	5.66 ± 2.16	3.50 ± 1.59	0.90–18.0
Eos (%)	3.14 ± 0.95	3.20 ± 1.22	0.00–7.50
Bas (%)	0.32 ± 0.16	0.60 ± 0.10	0.00–1.50
RBC (10^6^/μL)	8.77 ± 0.97	8.30 ± 2.38	6.50–11.50
HGB (g/dL)	13.58 ± 1.18	12.47 ± 3.29	11.00–16.50
HCT (%)	38.80 ± 4.85	36.20 ± 10.14	35.0–55.0
MCV (fL)	44.18 ± 1.18	43.70 ± 0.40	41.0–55.0
MCH (pg)	15.54 ± 0.36	15.13 ± 0.42	13.0–18.0
MCHC (g/dL)	35.22 ± 1.52	34.57 ± 0.64	30.0–36.0
RDW-CV (%)	15.36 ± 0.88	15.10 ± 0.44	12.0–19.0
PLT (10^3^/μL)	565.40 ± 610.21	1002.67 ± 573.90	400–1600
MPV (fL)	6.34 ± 0.11	6.20 ± 0.61	4.00–6.20

At the end of the study, mice were euthanized. Major organs, including the kidney, spleen, lung, liver, and heart, were then collected, rinsed with PBS, and fixed in 4% formalin. Tissues were sectioned, stained with hematoxylin and eosin, and examined microscopically (Figure 9). No substantial histopathological abnormalities were detected in any organ from the compound **A** treated mice. Kidney samples exhibited normal glomerular and tubular structures without signs of necrosis, glomerular atrophy, or inflammation. In the spleen, well-defined architecture with intact white pulp, red pulp, and marginal zones was observed. Lung tissue showed no evidence of injury or fibrosis. Liver sections maintained typical hepatic architecture with clearly defined central veins, hepatocytes, sinusoids, and nuclei. Heart tissues displayed no signs of infarction, inflammatory infiltration, myocardial rupture, or necrosis.

**Figure 9:**
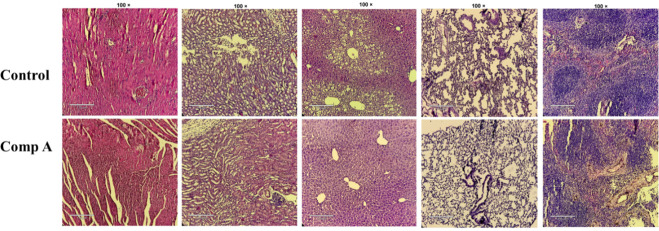
H&E-stained tissue sections of the kidney, spleen, lung, liver, and heart collected from mice after compound **A** treatment (scale bar, 100 μm).

Collectively, these results demonstrate that compound **A** does not induce toxicity in mice, even at a dose 10-fold higher than the therapeutic level. The absence of histological damage and stable hematological profiles indicate that compound **A** possesses a favorable safety profile for further development.

### Compound **A** Shows Great Translational Potential in Patient-Origin GBM Cells

To further evaluate the translational potential of compound **A**, it was examined with patient origin cell line DI318.^30^ The results exhibited in Figure 10 demonstrated that compound **A** inhibited the cell growth with an IC_50_ of 2.88 ± 0.03 μM, and decreased AR protein levels in a dose dependent manner. The results are consistent to the studies with the established GBM cells.

**Figure 10:**
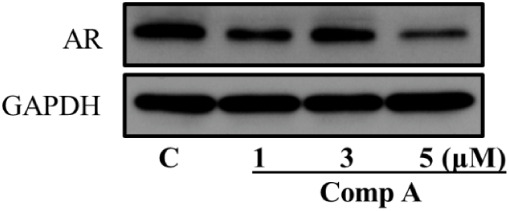
Compound **A** abolishes AR in DI318 patient origin cells. AR protein expression after compound treatment was assessed by Western blot analysis. Three independent experiments were performed.

## Conclusions

GBM is the most aggressive form of primary malignant brain tumor and is associated with poor survival.^2,31^ The disease occurs more frequently in men than in women, suggesting a possible sex-related component. AR has been implicated in GBM progression.^7,8^ HSP27 acts as a chaperone protein that stabilizes AR, and disruption of HSP27 represents a potential strategy to promote AR degradation in AR-overexpressing GBM.^24,25^ Our goal is to develop AR degraders as potential drug candidates that eliminate AR proteins in GBM, offering a mechanistic advantage over conventional AR antagonists. The lead compound does not bind directly to AR; instead, it targets HSP27, disrupting the HSP27–AR complex and thereby promoting AR degradation. This compound effectively inhibits the chaperone activity of HSP27, exhibits high potency and selectivity against AR-overexpressing GBM cells including established cell lines and patient origin cells, and induces degradation of both wild-type and mutant AR. *In vivo* studies demonstrate that compound **A** significantly suppresses U87 xenograft tumor growth and reduces AR levels in tumor tissues. Importantly, even at doses 10-fold higher than the effective antitumor dose, compound **A** shows no observable toxicity in mice. Pharmacokinetic profiling further reveals that compound **A** achieves a brain/plasma exposure of 52%, a level that not only surpasses TMZ but also represents a notably high degree of BBB penetration for small-molecule brain tumor therapeutics. Together, these *in vitro* and *in vivo* results establish compound **A** as a highly promising drug candidate for the treatment of AR-overexpressing GBM.

While the current *in vivo* efficacy study was conducted using a subcutaneous GBM xenograft model, this model does not fully recapitulate the complexity of an orthotopic brain tumor microenvironment. Although compound **A** demonstrates substantial brain penetration, further studies using intracranial GBM models will be necessary to more comprehensively evaluate its therapeutic efficacy within the brain, particularly in the context of the brain tumor barrier (BTB), tumor microenvironment, and regional drug distribution.

## Experimental Section

### Reagents

Thiazolyl Blue tetrazolium bromide, 98% (Alfa Aesar, P31B064). Insulin (Sigma-Aldrich, 91077C). α-Crystallin (Sigma-Aldrich, C4163). Dithiothreitol (DTT) (Amresco, EC# 222–468–7). DMEM (Cleveland Clinic Media Laboratory , 11–500p). RPMI 1640 (Cleveland Clinic, 10–500p). Neurobasal Medium, minus phenol red (ThermoFisher Scientific, 12348017). B-27 Supplement (50X), minus vitamin A (ThermoFisher Scientific, 12587010). Sodium pyruvate (ThermoFisher Scientific, 11360–070). Fibroblast growth factor (FGF, R&D Systems, 4114-TC-01M). Epidermal growth factor (EGF, R&D Systems, 236-EG-01M). l-Glutamine (ThermoFisher Scientific, 25030081). Geltrex (Gibco, A1413302). FBS (Atlanta Biologicals, S11150). Pen/Strep solution (Cleveland Clinic, 725–100p). Anti-β-actin antibody (Cell Signaling Technology, 4967S). DAPI (VWR, 95059–474). Anti-HSP27 antibody (Cell Signaling Technology, 2402S). Anti-Androgen Receptor antibody (Cell Signaling Technology, 5153S). Anti-AR-V7 antibody (Cell Signaling Technology, 68492S). Anti-Rabbit IgG (Cell Signaling Technology, 7074S). Anti-Rabbit Alexa Fluor 488 secondary antibody (Thermo Scientific, A-21206). Anti-Mouse Alexa Fluor 594 secondary antibody (Thermo Scientific, SA5–10168). Non-Fat dry milk (Rockland, B51–0500). Chemiluminescent Substrate (Thermo Scientific, 34577). All the other chemicals were of analytical grade.

### Cell Culture

The T98G and U87 cell lines were obtained from ATCC. Cells were grown in RPMI-1640 or DMEM supplemented with 10% fetal bovine serum (FBS), 100 U/mL penicillin, and 100 μg/mL streptomycin, and maintained at 37 °C in a humidified incubator with 5% CO_2_. DI318 human glioblastoma cells were isolated from a patient tumor sample at the Cleveland Clinic and maintained by the Lathia lab.^29^ Cells were cultured as adherent cells on Geltrex-coated plates in complete DI318 medium, consisting of Neurobasal medium without phenol red supplemented with B27 without vitamin A (2%), sodium pyruvate (1%), l-glutamine (1%), penicillin–streptomycin (1%), EGF (20 ng/mL), and FGF (20 ng/mL), and maintained at 37 °C in a humidified incubator with 5% CO_2_.

### Compound **A**

The synthetic procedure for compound **A** has been reported previously.^24,25^ The final product showed a purity greater than 99%. Chromatographic analysis was performed on a C18 column (2.0 mm × 150 mm, 5 μm; Phenomenex, Torrance, CA). H_2_O/CH_3_OH or H_2_O/CH_3_CN served as the mobile phase for isocratic elution at a flow rate of 0.2 mL/min. Samples were injected at a volume of 20 μL, and UV detection was monitored at 256 and 290 nm.

### Cell Viability Analysis

Cell viability was assessed using the MTT assay to determine the effect of compound **A** on T98G, U87, and DI318 cells, with eight replicate wells for each condition. Cells were seeded at a density of 3,000 cells per well in 96-well flat-bottom plates containing RPMI-1640, DMEM, or complete DI318 medium and allowed to attach for 24 h. The cells were then treated with different concentrations of compound **A** dissolved in DMSO (final DMSO concentration ≤0.1%) for 48 h, while control wells received the same amount of DMSO. Following treatment, cells were incubated with MTT solution (1 mg/mL in fresh medium, 100 μL per well) at 37 °C for 2 h. The medium was subsequently removed, and the resulting formazan crystals were dissolved in 200 μL of DMSO per well. Absorbance was measured at 570 nm using an ID3 microplate reader (Molecular Devices).

### Western Blotting

Cells were plated in 6-well culture plates and treated with either DMSO or compound **A**. After treatment, cells were lysed using RIPA buffer (Thermo Scientific, Prod# 89900) supplemented with a protease inhibitor cocktail (Thermo Scientific, Prod# 1861278). Lysates were kept on ice for 10 min and then transferred to 1.5 mL centrifuge tubes. Following centrifugation at 10,000*g* for 10 min, the supernatants were collected for further analysis. Protein concentrations were quantified using the Bradford Protein Assay kit (Bio-Rad). Equal amounts of protein (50 μg per sample) were mixed with 1× loading buffer and heated for 10 min. The samples were separated on a 10% SDS-polyacrylamide gel and transferred onto a PVDF membrane (Bio-Rad). Membranes were blocked for 2 h at room temperature in 5% nonfat milk prepared in TBS-T buffer (150 mM NaCl, 10 mM Tris, pH 7.4, 0.1% Tween-20), followed by overnight incubation with the primary antibody at 4 °C. After washing three times with TBS-T (10 min each wash), membranes were incubated with the appropriate secondary antibody for 1 h at room temperature. Membranes were washed again three times with TBS-T, and protein bands were visualized using SuperSignal West Pico Chemiluminescent Substrate (Pierce) according to the manufacturer’s instructions.

### HSP27 Chaperone Activity Assay

HSP27 chaperone activity was evaluated using an insulin aggregation assay. In each well of a 384-well plate, 24 μL of insulin solution (1 mg/mL) was combined with 3 μL of α-Crystallin (5 mg/mL), a functional fragment of HSP27 responsible for its chaperone activity, together with 71 μL PBS containing compound **A** (10 μM). The mixture was gently mixed and incubated at 37 °C for 5 min. Insulin aggregation was initiated by adding 2 μL of 1 M DTT prepared in water. Control reactions contained insulin with or without α-Crystallin in the presence of 0.1% DMSO. Aggregation of insulin was monitored by measuring absorbance at 400 nm every 3 min for 2 h using an ID3 microplate reader (Molecular Devices).

### Docking Study

The experimental structure for HSP27 (PDB ID: 6DV5) was downloaded from the RCSB Protein Data Bank. Chains A and B were used for further analysis. Nonterminal, unresolved regions were reconstructed using the MODELER web service from within UCSF ChimeraX.^31^ As some of the missing regions were of substantial length, 20 models were generated and used in the docking study. Docking of compound **A** was completed using AutoDock Vina 1.2.5.^32,33^ The search box (total volume 5,956 Å^3^) was centered on the catalytic region between chains A and B. A maximum of 10 poses were generated for each of the 20 models using an exhaustiveness of 480, with the highest scored pose (−7.9033 kcal/mol) selected for further analysis. Visualization of the docked pose was done in ChimeraX, while the 2-dimensional interaction diagram was generated in Schrödinger Maestro Viewer (version 2025–2).^31^

### Experimental Animals

Male C57BL/6 mice and nude mice were obtained from Taconic Laboratories (NY, USA). Animals were maintained in Plexiglas cages under controlled temperature and humidity with a 12 h light/dark cycle and had free access to food and water. All procedures involving animals followed the guidelines of the Cleveland State University Institutional Animal Care and Use Committee (IACUC) and were conducted under approved protocols (Ethics Approval Numbers: 21181-SUB-AS and 21199-SUB-AS).

### *In Vivo* Xenograft Study

All animal procedures were reviewed and approved by the IACUC. Male NCRNU nude mice (5–6 weeks old) were purchased from Taconic Laboratories (NY, USA). U87 cells were suspended in sterile PBS (100 μL), and 5 × 10^6^ cells were injected subcutaneously into both the left and right flanks of each mouse (two tumors per mouse; *n* = 4 per group, eight tumors per group). Tumor size and body weight were recorded three times per week using a vernier caliper. Tumor volume was calculated using the formula *V* = (2/3) × *d*_1_ × *d*_2_^2^, where *d*_1_ represents the larger diameter and *d*_2_ represents the smaller diameter. Once tumor volumes reached approximately 50 mm^3^, mice were assigned to receive either vehicle (DMSO) or compound **A** (20 mg/kg in PBS) by intraperitoneal injection three times per week for 14 days. Tumor growth was monitored throughout the treatment period. At the end of the study, mice were euthanized by CO_2_ exposure. Tumors were excised and weighed. Tumor tissues from each mouse were then pooled, homogenized in RIPA buffer supplemented with protease inhibitors and PMSF, and processed to obtain tumor lysates.

### Maximum Tolerated Dose (MTD) Study

Eight C57BL/6 mice were randomly assigned to two groups. Animals received daily IP of either vehicle (DMSO) or compound **A** (200 mg/kg prepared in 10% PEG400/PBS). After 10 days of treatment, the mice were euthanized for further analysis.

#### Hematological Analysis

Immediately after euthanasia, blood samples were collected from the heart for hematological examination. Blood parameters were measured using an Element HT5 hematology analyzer (Heska Corporation, USA).

#### Hematoxylin and Eosin (H&E) Staining

Heart, liver, spleen, lung, and kidney tissues were harvested and fixed in 10% neutral buffered formalin (approximately 10 mL fixative per cm^3^ of tissue). After fixation, tissues were sectioned and placed into embedding cassettes, followed by dehydration through graded ethanol solutions. Residual ethanol was removed with xylene before infiltration with paraffin wax. The samples were embedded in paraffin, and 4 μm sections were prepared using a microtome. Sections were subsequently stained with hematoxylin and eosin (H&E) for histological evaluation.

### Pharmacokinetic Study

For pharmacokinetic analysis, mice received compound **A** at a dose of 20 mg/kg via IP administration. At predetermined time points (0.083, 0.25, 0.5, 1, 2, 4, 6, 12, and 24 h), mice were sacrificed and blood samples (400 μL) were collected via cardiac puncture. Following blood collection, animals were perfused with PBS and brain tissues were harvested. Blood samples were centrifuged at 3,000*g* for 10 min at 4 °C to obtain plasma, which was stored at −80 °C until analysis. Brain tissues were weighed and homogenized in PBS (1:5, w/v) using a Dounce tissue grinder (DWK Life Sciences) on ice. Homogenates were aliquoted and stored at −80 °C prior to analysis.

### Determination of Compound **A** in Mouse Plasma and Brain

#### HPLC-MS/MS Conditions

Quantification of compound **A** in mouse plasma and brain samples was carried out using an HPLC-MS/MS system consisting of a Shimadzu UPLC instrument (Columbia, MD) equipped with a Prominence DGU-20A3R inline degasser, two LC-30AD pumps, a SIL-30AC autosampler, and a CBM-20A controller. Chromatographic separation was achieved on a Kinetex C18 column (50 mm × 2.1 mm, 1.3 μm). The mobile phase consisted of acetonitrile containing 0.1% formic acid and water (50:50, v/v) delivered at a flow rate of 0.3 mL/min. The column temperature was maintained at 36 °C, and the injection volume was set at 5 μL. Mass spectrometric detection was performed using an AB Sciex Qtrap 5500 mass spectrometer (Toronto, Canada) operated in negative electrospray ionization mode (ESI^–^). Quantification was conducted using multiple reaction monitoring (MRM). The ion transitions monitored were *m*/*z* 381.3→152.0 for compound A and *m*/*z* 499.1→267.0 for the internal standard (IS). The optimized ion source settings were as follows: ion spray voltage 4200 V, ion source temperature 450 °C, nebulizing gas 40 psi, auxiliary gas 40 psi, and curtain gas 45 psi.

The compound-specific parameters were set as follows. For compound **A**: declustering potential 40 V, entrance potential 5 V, collision energy 23 V, and collision entrance potential 15 V. For the IS: declustering potential 100 V, entrance potential 10 V, collision energy 35 V, and collision entrance potential 15 V.

#### Preparation of Standards and Samples

Stock solutions of compound **A** and IS were prepared in methanol at a concentration of 1.0 mg/mL. Working calibration standards of compound **A** were generated by serial dilution of the stock solution in methanol to obtain concentrations of 1.0, 2.0, 5.0, 10, 20, 50, 100, 200, 500, and 1000 ng/mL. A working solution of the IS (500 ng/mL) was prepared from its stock solution using methanol. All solutions were stored at 4 °C in the dark until use. Sample preparation was performed using a protein precipitation procedure. Mouse plasma or brain homogenate samples (brain tissue homogenized at 0.4 g in 2 mL PBS) were processed by mixing 100 μL of sample with 40 μL of the IS working solution (500 ng/mL) and 800 μL methanol in a 1.5 mL tube. After vortex mixing, the samples were centrifuged at 12,000*g* for 5 min. The resulting supernatant was transferred to a fresh tube and evaporated to dryness under a nitrogen stream. The residue was stored at −80 °C and subsequently reconstituted in 100 μL of 50% acetonitrile prior to analysis. Pharmacokinetic parameters were calculated using a noncompartmental analysis approach.

### Statistical Analysis

Statistical analyses and graphical representations were generated using GraphPad Prism (GraphPad Software Inc.) and Microsoft Excel (Microsoft Corporation). Densitometric analysis of Western blot bands was conducted with Quantity One software (Bio-Rad). IC_50_ values were calculated by nonlinear regression analysis. Differences between groups were evaluated using a two-tailed unpaired Student’s *t* test, and statistical significance was defined at a 95% confidence level.

Pharmacokinetic parameters were estimated using noncompartmental analysis in Excel (Microsoft 365, Microsoft Corp., Redmond, WA) and GraphPad Prism software (GraphPad Software Incorporated). Maximum concentration (*C*_max_) was obtained directly from observed data. The area under the concentration–time curve from time zero to the last measurable concentration (AUC_0–24h_) was calculated using the linear trapezoidal method. The half-life (*t*_1/2_) was calculated as ln(2)/the terminal elimination rate constant (λz). The terminal elimination rate constant (λz) was determined by log–linear regression of the terminal phase. AUC_0‑∞_ was calculated as AUC_0–24h_ + C_last/λz. Goodness of fit for terminal phase estimation was assessed using the coefficient of determination (*r* ^2^).

## Abbreviations

AR: androgen receptor
ASO: antisense oligonucleotides
BBB: blood–brain barrier
COX-2: cyclooxygenase 2
DTT: dithiothreitol
FBS: fetal bovine serum
GBM: Glioblastoma
HSP27: heat shock protein 27 kDa
IP: intraperitoneal injection
SiRN: short interfering RNA
TMZ: Temozolomide
